# PANoptosis, an indicator of COVID-19 severity and outcomes

**DOI:** 10.1093/bib/bbae124

**Published:** 2024-03-28

**Authors:** Qingyuan Yang, Wanmei Song, Hanizaier Reheman, Dan Wang, Jieming Qu, Yanan Li

**Affiliations:** Department of Pulmonary and Critical Care Medicine, Ruijin Hospital, Shanghai Jiao Tong University School of Medicine, Shanghai 200025, China; Institute of Respiratory Diseases, Shanghai Jiao Tong University School of Medicine, Shanghai 200025, China; Shanghai Key Laboratory of Emergency Prevention, Diagnosis and Treatment of Respiratory Infectious Diseases, Shanghai 200025, China; Department of Pulmonary and Critical Care Medicine, Ruijin Hospital, Shanghai Jiao Tong University School of Medicine, Shanghai 200025, China; Institute of Respiratory Diseases, Shanghai Jiao Tong University School of Medicine, Shanghai 200025, China; Shanghai Key Laboratory of Emergency Prevention, Diagnosis and Treatment of Respiratory Infectious Diseases, Shanghai 200025, China; Department of Pulmonary and Critical Care Medicine, Ruijin Hospital, Shanghai Jiao Tong University School of Medicine, Shanghai 200025, China; Institute of Respiratory Diseases, Shanghai Jiao Tong University School of Medicine, Shanghai 200025, China; Shanghai Key Laboratory of Emergency Prevention, Diagnosis and Treatment of Respiratory Infectious Diseases, Shanghai 200025, China; Shanghai Institute of Hematology, State Key Laboratory of Medical Genomics, National Research Center for Translational Medicine at Shanghai, Ruijin Hospital, Shanghai Jiao Tong University School of Medicine, Shanghai 200025, China; Department of Pulmonary and Critical Care Medicine, Ruijin Hospital, Shanghai Jiao Tong University School of Medicine, Shanghai 200025, China; Institute of Respiratory Diseases, Shanghai Jiao Tong University School of Medicine, Shanghai 200025, China; Shanghai Key Laboratory of Emergency Prevention, Diagnosis and Treatment of Respiratory Infectious Diseases, Shanghai 200025, China; Department of Pulmonary and Critical Care Medicine, Ruijin Hospital, Shanghai Jiao Tong University School of Medicine, Shanghai 200025, China; Institute of Respiratory Diseases, Shanghai Jiao Tong University School of Medicine, Shanghai 200025, China; Shanghai Key Laboratory of Emergency Prevention, Diagnosis and Treatment of Respiratory Infectious Diseases, Shanghai 200025, China

**Keywords:** PANoptosis, COVID-19, single-cell RNA-seq, bulk RNA-seq

## Abstract

Coronavirus disease 2019 (COVID-19) has been wreaking havoc for 3 years. PANoptosis, a distinct and physiologically relevant inflammatory programmed cell death, perpetuates cytokine storm and multi-organ injuries in COVID-19. Although PANoptosis performs indispensable roles in host defense, further investigation is needed to elucidate the exact processes through which PANoptosis modulates immunological responses and prognosis in COVID-19. This study conducted a bioinformatics analysis of online single-cell RNA sequence (scRNA-seq) and bulk RNA-seq datasets to explore the potential of PANoptosis as an indicator of COVID-19 severity. The degree of PANoptosis in bronchoalveolar lavage fluid (BALF) and peripheral blood mononuclear cells (PBMC) indicated the severity of COVID-19. Single-cell transcriptomics identified pro-inflammatory monocytes as one of the primary sites of PANoptosis in COVID-19. The study subsequently demonstrated the immune and metabolic characteristics of this group of pro-inflammatory monocytes. In addition, the analysis illustrated that dexamethasone was likely to alleviate inflammation in COVID-19 by mitigating PANoptosis. Finally, the study showed that the PANoptosis-related genes could predict the intensive care unit admission (ICU) and outcomes of COVID-19 patients who are hospitalized.

## INTRODUCTION

Coronavirus disease 2019 (COVID-19), caused by severe acute respiratory syndrome coronavirus 2 (SARS-CoV-2), has been wreaking havoc for 3 years and has claimed more than 6.9 million lives, according to the World Health Organisation (WHO). The innate and adaptive immune responses orchestrate the host defense against COVID-19. The initial defense mechanism against SARS-CoV-2 involves the detection of pathogens through a variety of pattern recognition receptors (PRRs). Following the activation of PRRs, subsequent signaling pathways lead to the release of cytokines. Among these cytokines, type I and III interferons (IFNs) are considered to be of paramount importance [1, 2], as they are responsible for initiating the antiviral program and augmenting the adaptive immune reaction [2]. SARS-CoV-2-elicited CD4^+^ and CD8 T^+^ cells target virus’s antigenic epitopes and coordinate with antibodies to perform antiviral functions [3]. Notably, the lethal outcomes in COVID-19 are primarily attributed to the failure in mounting a prompt and effective immune response, as well as the inability to regulate excessive immune reactions [2]. Although the direct virus-mediated tissue damage and the subsequent local and systematic inflammatory response constitute the two phases of the progression of pneumonia caused by SARS-CoV-2, the concrete mechanism remains to be elucidated.

PANoptosis, driven by TNF-α and IFN-γ, perpetuates cytokine storm and multi-organ injuries in COVID-19 [4, 5]. PANoptosis is defined as a distinct and physiologically significant form of inflammatory programmed cell death (PCD) that is controlled by the PANoptosome complex, exhibiting hallmark traits of pyroptosis, apoptosis, and necroptosis [6]. The formation of gasdermin (GSDM)-dependent pores on the plasma membrane, subsequent to the inflammasomes’ assembly and activation, stands as a hallmark of pyroptosis [7]. Apoptosis is carried out by caspase-3 and -7 after the activation of upstream initiator caspases, caspase-8/10 or caspase-9 [8]. Necroptosis is a lytic form of cell death, defined by the activation of the kinases RIPK1 and RIPK3 [9]. PANoptosis is an integrated biological process based on the extensive interactions among PCDs rather than the mere addition of pyroptosis, apoptosis and necroptosis. The PANoptosome serves as a molecular framework for the key upstream molecules of the abovementioned three PCD pathways to activate pro-inflammatory cell death [10]. RIPK1, ASC, NLRP3 and caspase-8 were the components of PANoptosome to be initially identified [11]. Subsequent investigations have demonstrated that RIPK3, caspase-6, ZBP1 and caspase-1 are additional constituents of the PANoptosome [12, 13]. A wide range of pathogens, such as bacteria, viruses and fungi, have the capability to trigger PANoptosis in cells [13–15]. It is anticipated that proper activation of PANoptosis will be a promising therapeutic approach for treating pathogenic infections since it stimulates immune cell infiltration to eradicate pathogens [16].

Although PANoptosis performs indispensable roles in host defense, more research is required to ascertain the precise mechanisms by which PANoptosis modulates immunological responses and prognosis in COVID-19. This work conducted the analysis of online single-cell RNA sequence (scRNA-seq) and bulk RNA-seq datasets to explore the potential of PANoptosis as the indicator of COVID-19 severity. The extent of PANoptosis was found to have a positive correlation with the disease severity. Moreover, dexamethasone therapy could mitigate the effects of PANoptosis. In addition, we have identified the primary site of PANoptosis to be monocytes, particularly the pro-inflammatory monocyte subcluster, and illustrated the immunological and metabolic properties of pro-inflammatory monocytes. Furthermore, the lasso regression model generated using PANoptosis genes of two bulk RNA seq datasets distinguished severe COVID-19 patients requiring intensive care unit (ICU) care from non-severe hospitalized cases accurately. Therefore, this study contributed important knowledge regarding the role of PANoptosis in COVID-19 and its implications for diagnosis and treatment.

## MATERIALS AND METHODS

### Data collection

The single-cell RNA sequencing (scRNA-seq) data for COVID-19 analyzed in this study were sourced from the Gene Expression Omnibus (GEO) database (https://www.ncbi.nlm.nih.gov/geo/) with accession numbers GSE145926 (BALF) and GSE157789 (PBMC-Dex) [17, 18]. The dataset of PBMC [19] was obtained from https://covid19cellatlas.org/. The bulk RNA sequencing (RNA-seq) data utilized in this research were obtained from the GEO database, with the accession numbers GSE157103 [20] and GSE215865 [21].

In pursuit of understanding the impact of PANoptosis on COVID-19 at the genetic level, this study excluded transcriptome sequencing data from five patients with bacterial acute respiratory distress syndrome (ARDS) in the PBMC-Dex dataset and removed five samples from hospitalized non-COVID-19 along with 12 samples from individuals treated with intravenous lipopolysaccharide (IV-LPS) in the PBMC dataset. In addition, we merged the samples of the severe and critical COVID-19 patients from the PBMC datasets into a single severe group to make the disease severity category in the PBMC dataset consistent with that in the BALF dataset, as the severe group in the BALF dataset included patients with severe/critical infection.

We selected specific samples from the GSE157103 and GSE215865 datasets to investigate the diagnostic potential of PANoptosis for COVID-19 disease progression. Our analysis included leukocyte samples from 100 COVID-19 patients in the GSE157103 dataset. In addition, we incorporated whole blood samples from the GSE215865 dataset, focusing on individuals with moderate disease severity (*n* = 172) and severe cases with end-organ damage (*n* = 45) at Days_Since_First_Sample = 0.

### Data preprocessing

For each cell from all patients and healthy controls, the criteria set were: a gene count ranging from 200 to 6000, a UMI (Unique Molecular Identifier) count between 500 and 1000, and a mitochondrial gene percentage of <0.1. The filtered gene-barcode matrix of each sample was integrated using the merge function available in Seurat (v4.2.0) [22]. The first 20 dimensions of principal component analysis (PCA) were employed in parameter settings. Using default parameters, the NormalizeData() function in Seurat (v4.2.0) was used to first normalize the filtered gene-barcode matrix. After scaling the data (using the ScaleData function), the top 2000 variable genes were found using the default parameters of the Seurat FindVariableFeatures function.

Using the top 2000 variable genes, PCA was conducted. Using Harmony (v0.1.0) [23], batch effects were removed across different donors. Next,uniform manifold approximation and projection (UMAP) analysis was conducted on the harmony embeddings to visualize the cells. Simultaneously, graph-based clustering was carried out on the harmony-reduced data for cluster analysis using Seurat (v4.2.0). A resolution of 2 was chosen to achieve a finer result. Marker genes for each cluster were determined using FindAllMarkers() function. Subsequently, clusters were annotated and subsetted into major cellular lineages based on the expression of canonical marker genes, employing the subset() function. These included EPECAM, KRT19, PROM1 and ALDH1A1 for epithelial cells; S100A8, CD163 and CD14 for monocytes; CD1E and CD1C for myeloid dendritic cells (mDCs); CLEC4C and CLIC3 for plasmacytoid dendritic cells (pDCs); KLRB1, NCR1, GNLY, CD3D, CD3E, CD8A, CD8B, CD4 and IL7R for T and natural killer (NK) cells; CD79A, CD79B and MS4A1 for B cells; MZB1 and SDC1 for plasma cells; PF4, PPBP and GP9 for platelets; CD34, EMCN and THY1 for hematopoietic stem cells (HSCs); and HBA2, HBB and HBD for red blood cells (RBCs). Individual sub-clustering was then performed for the monocyte subsets.

### Calculate module scores for gene sets expression programs

PANoptosis score was calculated by AddModuleScore() from Seurat (v4.2.0) and UCell (v 2.2.0) [24], which considered a list of PANoptosis key genes (ZBP1, NLRP3, PYCARD, CASP1, CASP8, FADD, RIPK1, RIPK3, GSDMD, CASP3, CASP6, CASP7 and MLKL) to determine the average expression levels of each cell type or group on a single-cell level.

### Differential gene expression analysis at the single-cell level

Differential gene expression analysis was performed using the Seurat FindAllMarkers function with default parameters. Genes were deemed differentially expressed (DEGs) if they showed statistical significance with an adjusted *P*-value <0.05 and an absolute log2-fold change (|Log2Foldchange|) ≥1.

### Gene functional annotation

For DEGs, Gene Ontology (GO) and Kyoto Encyclopedia of Genes and Genomes (KEGG) analyses were conducted using clusterProfiler [25]. This tool was employed for statistical analysis and visualization of functional profiles associated with genes and gene clusters.

### Quantifying the single-cell metabolism activity

To quantify the metabolic activity of pro-inflammatory monocytes, the R package scMetabolism (v 0.2.1) [26] was utilized, employing the sc.metabolism.Seurat() function. For this analysis, we specified the metabolism type as ‘KEGG’. In addition, we utilized two distinct methods, namely, AUCell and VISION, to assess metabolic activity.

### Bulk RNA-sequencing data analysis

Two bulk RNA-sequencing datasets (GSE157103 and GSE215865) were collected for our study. We focused on 13 PANoptosis genes (ZBP1, NLRP3, PYCARD, CASP1, CASP8, FADD, RIPK1, RIPK3, GSDMD, CASP3, CASP6, CASP7, MLKL) as potential predictors. Lasso regression was employed as modeling technique. The 13 PANoptosis genes were used as input features and the target variables (NON-ICU/Moderate, ICU/Severe) as the response variable in the lasso regression model. To evaluate the stability and generalizability of our model, we implemented *k*-fold cross-validation with *k* = 5 and times = 5. In each fold, the dataset was stratified and randomly partitioned into training (80% of the data) and testing sets (20% of the data). The model was then trained on the training set and assessed for performance on the testing set. The results from each fold were then averaged to provide a more robust evaluation of the model’s performance. The cross-validation results were aggregated to provide an overall evaluation of the model’s predictive accuracy and stability. This repeated cross-validation technique was particularly useful in providing a more robust and reliable estimate of model accuracy, reducing the likelihood of performance estimation biases due to random sampling.

Furthermore, to better demonstrate the classifiability of the PANoptosis genes, we chose a random set of genes with an equal number of PANoptosis genes of these two bulk RNA-seq datasets, respectively, to construct a lasso regression model and perform *k*-fold cross-validation as described above.

Subsequently, we compared the classification performance of these randomly chosen gene sets with that of the PANoptosis genes.

### Statistical analysis

The Student *t*-test was conducted using ggpubr (v0.4.0) to calculate the *P*-value between two groups. Kruskal–Wallis tests, also implemented with ggpubr (v0.4.0), were utilized to identify overall significant differences at the population level. Correlation plots were generated using the R package corrplot (v0.94), available at https://github.com/taiyun/corrplot/.

## RESULTS

### PANoptosis increased significantly in COVID-19 and was related to the inflammatory response

**Figure 1:**
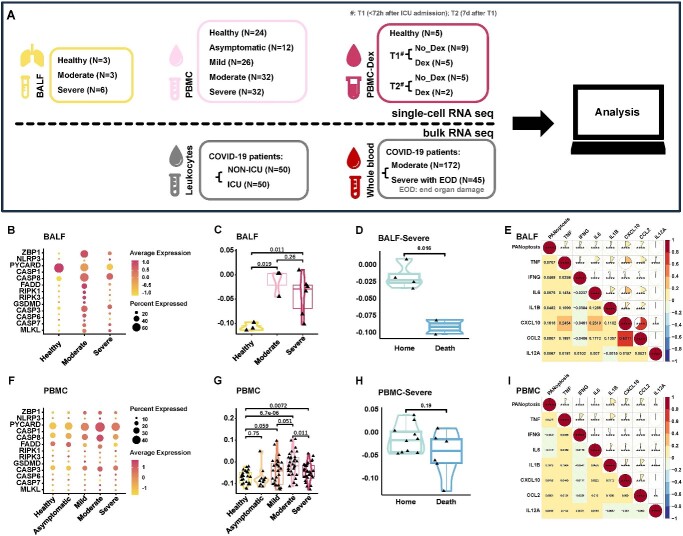
PANoptosis increased significantly in COVID-19 and was related to the inflammatory response. (**A**) Workflow of this study. Three single-cell RNA-seq datasets were used: the BALF dataset included healthy controls (*N* = 3) and patients with moderate (*N* = 3) and severe (*N* = 6) COVID-19; the PBMC dataset involved healthy controls (*N* = 24) and patients with asymptomatic (*N* = 12), mild (*N* = 26), moderate (*N* = 32) and severe (*N* = 32) COVID-19; the PBMC-Dex dataset compromised healthy controls (*N* = 5) and COVID-19 ARDS patients, both non-dexamethasone-treated (*N* = 9 at T1 and *N* = 5 at T2) and dexamethasone-treated (*N* = 5 at T1 and *N* = 2 at T2), who were admitted to the ICU. T1 denotes the first blood draw within 72 h of ICU admission, whereas T2 represents a follow-up blood draw taken 7 days after T1. In addition, two bulk RNA sequencing datasets were employed: one dataset compromised NON-ICU (*N* = 50) and ICU (*N* = 50) COVID-19 patients; while the other dataset included moderate (*N* = 172) and severe with end-organ damage (*N* = 45) COVID-19 patients. (**B**) Expression levels of key PANoptosis genes in all cells from COVID-19 patients and healthy controls within the BALF dataset, categorized by disease severity. (**C**) PANoptosis scores for all cells from COVID-19 patients and healthy controls within the BALF dataset, categorized by disease severity (using two-sided Student’s *t*-test for pairwise comparisons). The PANoptosis score was computed using the AddModuleScore() function from Seurat (v4.2.0). (**D**) PANoptosis scores for all cells from severe COVID-19 patients within the BALF dataset, categorized by outcome (using two-sided Student’s *t*-test for pairwise comparisons). The PANoptosis score was computed using the AddModuleScore() function from Seurat (v4.2.0). (**E**) Heatmap displaying the correlation between PANoptosis scores and inflammatory cytokines in all cells from both COVID-19 patients and healthy controls within the BALF dataset. Spearman correlation coefficients and exact two-sided *P*-values are shown. The PANoptosis score was computed using the AddModuleScore() function from Seurat (v4.2.0). (**F**) Expression levels of key PANoptosis genes in all cells from COVID-19 patients and healthy controls within the PBMC dataset, categorized by disease severity. (**G**) PANoptosis scores for all cells from COVID-19 patients and healthy controls within the PBMC dataset, categorized by disease severity (using two-sided Student’s *t*-test for pairwise comparisons). The PANoptosis score was computed using the AddModuleScore() function from Seurat (v4.2.0). (**H**) PANoptosis scores for all cells from severe COVID-19 patients within the PBMC dataset, categorized by outcome (using two-sided Student’s *t*-test for pairwise comparisons). The PANoptosis score was computed using the AddModuleScore() function from Seurat (v4.2.0). (**I**) Heatmap displaying the correlation between PANoptosis scores and inflammatory cytokines in all cells from COVID-19 patients and healthy controls within the PBMC dataset. Spearman correlation coefficients and exact two-sided *P*-values are shown. The PANoptosis score was computed using the AddModuleScore() function from Seurat (v4.2.0). Significance values: ns (not significant), *P* > 0.05, ^*^P < 0.05, ^*^^*^P < 0.01, ^*^^*^^*^P < 0.001, ^*^^*^^*^^*^P < 0.0001.

PANoptosis perpetuates cytokine storm and multi-organ injuries in COVID-19 [14]. The precise mechanism by which PANoptosis mediates the pathogenic process of COVID-19 has yet to be definitively established. We investigated the occurrence of PANoptosis in different severity groups by analyzing the scRNA sequencing data from bronchoalveolar lavage fluid (BALF) and peripheral blood mononuclear cells (PBMCs), and bulk RNA sequencing data of leukocytes and whole blood (Figure 1A). The PANoptosis elevated in both the BALF and PBMC of COVID-19 cases (Figure 1B–C, F–G; Supplementary Figure 1A and H). The PANoptosis showed a positive correlation with disease severity, ranging from the healthy group to moderate COVID-19 (Supplementary Figure 1B–C, I–J). However, there was a negative correlation between the occurrence of PANoptosis and disease severity when transitioning from moderate to severe COVID-19 (Supplementary Figure 1D–E, K–L). These results suggested that the degree of PANoptosis peaked in moderate COVID-19, and PANoptosis downregulated in severe COVID-19. Furthermore, a significant reduction in PANoptosis was observed in both the BALF and PBMC of severe patients who did not survive hospitalization (Figure 1D and H; Supplementary Figure 1F and M). These findings suggested a potential association between a poorer clinical outcome and decreased PANoptosis in severe COVID-19 patients. During the advanced stages of the disease, when the immune system becomes exhausted, there are limited cells remaining to execute immune defense responses through the initiation of PANoptosis.

In COVID-19, there is an increase in inflammatory cytokines, which are associated with cytokine storms [1]. The levels of TNF, IL-1β, CXCL10 and CCL2 were significantly positively correlated with PANoptosis in both BALF and PBMC (Figure 1E and I; Supplementary Figure 1G and N), suggesting a strong association between PANoptosis and the inflammatory response. It is noteworthy that the release of inflammatory cytokines and PANoptosis exhibit reciprocal causation: the synergism of TNF-α and IFN-γ initiates PANoptosis in COVID-19, and PANoptosis, in turn, triggers cytokine storm [4].

### Pro-inflammatory monocytes were one of the main sites of PANoptosis

**Figure 2:**
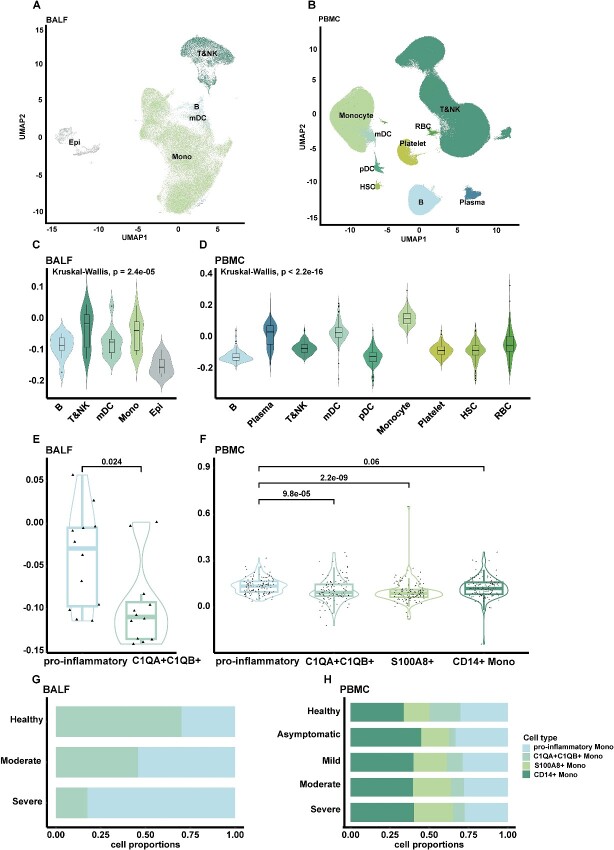
PANoptosis mainly occurred in pro-inflammatory monocytes. (**A**) UMAP embedding illustrating all cells from COVID-19 patients and healthy controls within the BALF dataset. A total of 72 174 cells were plotted from 12 biologically independent samples. (**B**) UMAP embedding displaying all cells from COVID-19 patients and healthy controls within the PBMC dataset, with colors indicating manual cell type annotation. A total of 619 008 cells were plotted from 126 biologically independent samples. (**C**) PANoptosis scores for each cell subpopulation from COVID-19 patients and healthy controls within the BALF dataset. Exact *P*-values from the Kruskal–Wallis test are shown. The PANoptosis scores was calculated using the AddModuleScore() function from Seurat (v4.2.0). (**D**) PANoptosis scores for each cell subpopulation from COVID-19 patients and healthy controls within the PBMC dataset. Exact *P*-values from the Kruskal–Wallis test are shown. The PANoptosis score was computed using the AddModuleScore() function from Seurat (v4.2.0). (**E**) PANoptosis scores for different monocyte subsets from COVID-19 patients and healthy controls within the BALF dataset. Exact *P*-values were determined using Student’s *t*-test. The PANoptosis score was computed using the AddModuleScore() function from Seurat (v4.2.0). (**F**) PANoptosis scores for different monocyte subsets from COVID-19 patients and healthy controls within the PBMC dataset. Exact *P*-values were determined using Student’s *t*-test. The PANoptosis score was computed using the AddModuleScore() function from Seurat (v4.2.0). (**G**) Proportion of monocyte subsets in each disease severity category within the BALF dataset. (**H**) Proportion of monocyte subsets in each disease severity category within the PBMC dataset. Significance values: ns (not significant), *P* > 0.05, ^*^P < 0.05, ^*^^*^P < 0.01, ^*^^*^^*^P < 0.001, ^*^^*^^*^^*^P < 0.0001.

To further investigate whether PANoptosis in COVID-19 exhibits a cell-type preference, we classified cells from the BALF and PBMC datasets into different cell types based on their marker genes (Figure 2A and B, Supplementary Figure 4A–D) after using harmony (v0.1.0) to remove batch effects across different donors (Supplementary Figure 2A and B; Supplementary Figure 3A and B). In BALF, the PANoptosis score was highest in T cells, NK cells and monocytes (Figure 2C, Supplementary Figure 4E). In PBMC, monocytes displayed the highest PANoptosis scores (Figure 2D, Supplementary Figure 4F). Given the consistent high PANoptosis scores observed in monocytes in both BALF and PBMC, we focused on monocytes and further subdivided them (Supplementary Figure 4G–J). It turned out that pro-inflammatory monocytes serve as one of the primary sites for PANoptosis (Figure 2E and F, Supplementary Figure 4K and L).

In BALF, there was a progressive increase in the proportion of pro-inflammatory monocytes in alignment with the disease severity (Figure 2G). Conversely, an opposing pattern was observed in PBMC (Figure 2H). This phenomenon can be attributed to the migration of pro-inflammatory monocytes from the bloodstream to the lung to carry out their immune defense functions during COVID-19 infection. These results demonstrated that pro-inflammatory monocytes were the primary executors of PANoptosis, and they migrated from the blood to the lungs in COVID-19.

### The PANoptosis extent in pro-inflammatory monocytes reflected the disease severity and inflammatory conditions

**Figure 3:**
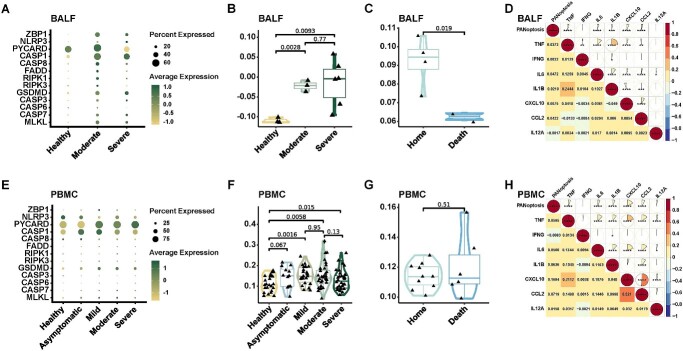
The extent of PANoptosis in pro-inflammatory monocytes reflected the disease severity and inflammatory conditions. (**A**) Expression levels of key PANoptosis genes in pro-inflammatory monocytes from both COVID-19 patients and healthy controls within the BALF dataset, categorized by disease severity. (**B**) PANoptosis scores of pro-inflammatory monocytes from COVID-19 patients and healthy controls within the BALF dataset, categorized by disease severity (using two-sided Student’s *t*-test for pairwise comparisons). The PANoptosis score was computed using the AddModuleScore() function from Seurat (v4.2.0). (**C**) PANoptosis scores of pro-inflammatory monocytes from severe COVID-19 patients within the BALF dataset, categorized by outcome (using two-sided Student’s *t*-test for pairwise comparisons). The PANoptosis score was computed using the AddModuleScore() function from Seurat (v4.2.0). (**D**) Heatmap illustrating the correlation between PANoptosis scores and inflammatory cytokines in pro-inflammatory monocytes from COVID-19 patients and healthy controls within the BALF dataset. Spearman correlation coefficients and exact two-sided *P*-values are shown. The PANoptosis score was computed using the AddModuleScore() function from Seurat (v4.2.0). (**E**) Expression levels of key PANoptosis genes of pro-inflammatory monocytes from COVID-19 patients and healthy controls within the PBMC dataset, categorized by disease severity. (**F**) PANoptosis scores of pro-inflammatory monocytes from COVID-19 patients and healthy controls within the PBMC dataset, categorized by disease severity (using two-sided Student’s *t*-test for pairwise comparisons). The PANoptosis score was computed using the AddModuleScore() function from Seurat (v4.2.0). (**G**) PANoptosis scores of pro-inflammatory monocytes from severe COVID-19 patients within the PBMC dataset, categorized by outcome (using two-sided Student’s *t*-test for pairwise comparisons). The PANoptosis score was computed using the AddModuleScore() function from Seurat (v4.2.0). (**H**) Heatmap illustrating the correlation between PANoptosis scores and inflammatory cytokines in pro-inflammatory monocytes from COVID-19 patients and healthy controls within the PBMC dataset. Spearman correlation coefficients and exact two-sided *P*-values are shown. The PANoptosis score was computed using the AddModuleScore() function from Seurat (v4.2.0). Significance values: ns (not significant), *P* > 0.05, ^*^P < 0.05, ^*^^*^P < 0.01, ^*^^*^^*^P < 0.001, ^*^^*^^*^^*^P < 0.0001.

Monocytes mediate the host antimicrobial defense and pathogenesis of inflammatory diseases [27]. To further substantiate pro-inflammatory monocytes as primary sites of PANoptosis in COVID-19, we explored their PANoptosis status. Consistently, in both BALF and PBMC, PANoptosis exhibited significant upregulation in pro-inflammatory monocytes in COVID-19 (Figure 3A–B, E–F; Supplementary Figure 5A and H). From the healthy group to moderate COVID-19, PANoptosis showed a positive correlation with disease severity (Supplementary Figure 5B–C, I–J). Notably, there was no statistically significant difference in the level of PANoptosis observed between moderate and severe COVID-19 in BALF (Supplementary Figure 5D and E). Nevertheless, in PBMC, a marginal decrease in PANoptosis was observed in the severe group compared to the moderate group (Supplementary Figure 5K and L). The positive correlation between PANoptosis and disease severity was more pronounced in BALF as opposed to PBMC (Supplementary Figure 5B–C, I–J), suggesting that PANoptosis in BALF could better reflect the state of illness.

Furthermore, a decrease in PANoptosis was observed in severe patients who passed away, both in BALF and PBMC (Figure 3C and G; Supplementary Figure 5F and M), highlighting a correlation between reduced PANoptosis in severe COVID-19 patients and unfavorable clinical outcomes.

In pro-inflammatory monocytes, we also identified a significant positive correlation between PANoptosis and the levels of various inflammatory cytokines, including IL-6, IL-1β, CXCL10, CCL2 and TNF (Figure 3D and H; Supplementary Figure 5G and N). Generally, the findings observed in all cell types from BALF and PBMC (Figure 1B–I) were recapitulated in pro-inflammatory monocytes (Figure 3A–H), indicating that the extent of PANoptosis in pro-inflammatory monocytes could mirror disease severity.

### Dexamethasone therapy in COVID-19 suppressed PANoptosis

WHO strongly recommended corticosteroids for treating hospitalized patients with COVID-19 who require respiratory support, as corticosteroids effectively reduce the severity and mortality of most severely affected patients in a non-specific and general manner [28]. Nonetheless, there have been few studies investigating the potential of corticosteroids in regulating PANoptosis. Hence, we analyzed the PBMC-Dex dataset to explore the mechanism underlying the beneficial effects of dexamethasone in COVID-19 (Supplementary Figure 6, Supplementary Figure 7A–D). The PBMC-Dex dataset included samples collected at two different timepoints: T1 samples were obtained within 72 h of admission to the ICU, whereas T2 samples were collected 7 days after T1. A total of 5 healthy controls, 14 non-dexamethasone COVID-19 ARDS (*n* = 9 at T1 and *n* = 5 at T2) and 7 dexamethasone-treated COVID-19 ARDS (*n* = 5 at T1 and *n* = 2 at T2) patients admitted to the ICU were included in the PBMC-Dex dataset [18]. All patients in the dexamethasone-treated group received dexamethasone (6 mg per day) upon hospital admission.

**Figure 4:**
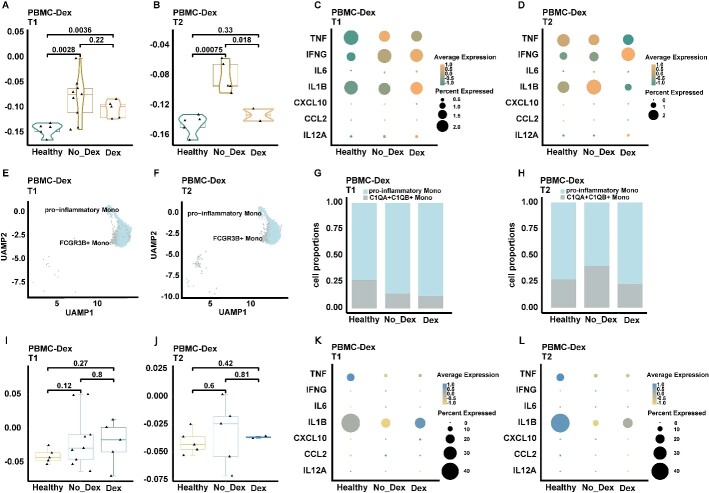
Dexamethasone therapy in COVID-19 suppressed PANoptosis. (**A**) PANoptosis scores of all cells from healthy controls and COVID-19 within the PBMC-Dex dataset at T1, categorized by treatment group (using two-sided Student’s *t*-test for pairwise comparisons). The PANoptosis score was computed using the AddModuleScore() function from Seurat (v4.2.0). (**B**) PANoptosis scores of all cells from healthy controls and COVID-19 within the PBMC-Dex dataset at T2, categorized by treatment group (using two-sided Student’s *t*-test for pairwise comparisons). The PANoptosis score was computed using the AddModuleScore() function from Seurat (v4.2.0). (**C**) Expression levels of inflammatory cytokines in all cells from healthy controls and COVID-19 within the PBMC-Dex dataset at T1, categorized by treatment group. (**D**) Expression levels of inflammatory cytokines in all cells from healthy controls and COVID-19 within the PBMC-Dex dataset at T2, categorized by treatment group. (**E**) UMAP embedding displaying monocytes from healthy controls and COVID-19 within the PBMC-Dex dataset at T1. A total of 3202 cells were plotted from 19 biologically independent samples. (**F**) UMAP embedding illustrating monocytes from healthy controls and COVID-19 patients within the PBMC-Dex dataset at T2. A total of 3027 cells were plotted from 12 biologically independent samples. (**G**) Proportion of monocyte subsets in different treatment groups at T1 within the PBMC-Dex dataset. (**H**) Proportion of monocyte subsets in different treatment groups at T2 within the PBMC-Dex dataset. (**I**) PANoptosis scores of pro-inflammatory monocytes from healthy controls and COVID-19 patients at T1 within the PBMC-Dex dataset, categorized by treatment group. Exact *P*-values from Student’s *t*-test are shown. The PANoptosis score was computed using the AddModuleScore() function from Seurat (v4.2.0). (**J**) PANoptosis scores of pro-inflammatory monocytes from COVID-19 patients at T2 within the PBMC-Dex dataset, categorized by treatment group. Exact *P*-values from Student’s *t*-test are shown. The PANoptosis score was computed using the AddModuleScore() function from Seurat (v4.2.0). (**K**) Expression levels of inflammatory cytokines of pro-inflammatory monocytes from COVID-19 patients at T1 within the PBMC-Dex dataset, categorized by treatment group. (**L**) Expression levels of inflammatory cytokines of pro-inflammatory monocytes from COVID-19 patients at T2 within the PBMC-Dex dataset, categorized by treatment group. Significance values: ns (not significant), *P* > 0.05, ^*^P < 0.05, ^*^^*^P < 0.01, ^*^^*^^*^P < 0.001, ^*^^*^^*^^*^P < 0.0001.

It was observed that the administration of dexamethasone resulted in a significant decrease in PANoptosis levels (Figure 4A and B, Supplementary Figure 7E and F) and a reduction in the cytokine storm (Figure 4C and D) at T2, collectively indicating the suppressive effect of dexamethasone. In addition, we investigated the impact of dexamethasone on pro-inflammatory monocytes (Figure 4E and F, Supplementary Figure 7G). At T1, dexamethasone treatment had a minimal impact on the proportion of pro-inflammatory monocytes (Figure 4G). However, at T2, there was a notable increase in the percentage of pro-inflammatory monocytes in the Dex group in comparison to the No-Dex group (Figure 4H), implying that dexamethasone may enhance the differentiation of monocytes into pro-inflammatory monocytes.

At T1, dexamethasone treatment did not yield a statistically significant reduction in PANoptosis levels in pro-inflammatory monocytes (Figure 4I, Supplementary Figure 7H). However, at T2, PANoptosis was observed to be downregulated in the dexamethasone-treated group (Figure 4J, Supplementary Figure 7I). The suppressive impact of dexamethasone on PANoptosis was more significant in the late phase of post-ICU admission (T2) (Figure 4A–B, I–J; Supplementary Figure 7E–F, H–I), demonstrating that the immunomodulatory action of corticosteroids may not manifest immediately.

Dexamethasone therapy not only downregulated the levels of various inflammatory factors in pro-inflammatory monocytes, such as IL-6, IL-1β, CXCL10 and CCL2 at T1 (Figure 4K), but also led to their upregulation at T2 in the Dex group (Figure 4L). This indicated that the therapeutic effect of dexamethasone extends beyond a mere reduction in pro-inflammatory factors and involves the alteration of the immune response landscape in the host.

### The immune and metabolic characteristics of pro-inflammatory monocytes in COVID-19 infection

**Figure 5:**
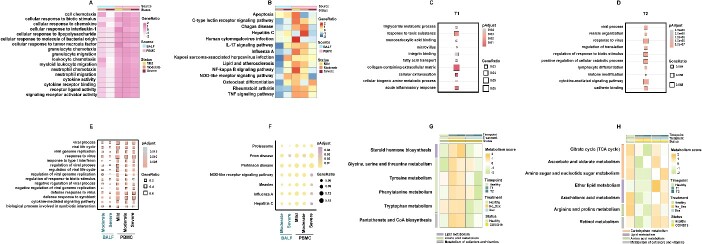
The immune and metabolic characteristics of pro-inflammatory monocytes in COVID-19 infection. (**A**–**B**) Heatmaps displaying the commonly upregulated Gene Ontology (GO) pathways (**A**) and Kyoto Encyclopedia of Genes and Genomes (KEGG) pathways (**B**) in pro-inflammatory monocytes compared to all other monocytes within each COVID-19 severity, separately for the BALF and PBMC datasets. All presented pathways achieved statistical significance at the *P* <0.05 confidence level, determined using Seurat’s implementation of the Wilcoxon rank-sum test. (**C**–**D**) Dot plots illustrating the top 10 specifically upregulated GO pathways in pro-inflammatory monocytes compared to all other monocytes within non-dexamethasone-treated COVID-19 ARDS at T1 (**C**) and T2 (**D**) within the PBMC-Dex dataset. (**E**–**F**) Dot plots displaying the commonly upregulated GO pathways (**E**) and KEGG pathways (**F**) in pro-inflammatory monocytes of each COVID-19 severity compared to pro-inflammatory monocytes from healthy controls, separately for the BALF and PBMC datasets. The color scale represents adjusted *P*-values. All displayed pathways achieved statistical significance at the *P* <0.05 confidence level, determined using Seurat’s implementation of the Wilcoxon rank-sum test. (**G**) Heatmap showing metabolic pathways that are upregulated in COVID-19 and subsequently downregulated after dexamethasone treatment of pro-inflammatory monocytes within the PBMC-Dex dataset. The heatmap color scale represents KEGG scores calculated using the R package scMetabolism with the ‘AUCell’ method. (**H**) Heatmap illustrating metabolic pathways that are downregulated in COVID-19 and subsequently upregulated after dexamethasone treatment of pro-inflammatory monocytes within the PBMC-Dex dataset. The heatmap color scale represents KEGG scores calculated using the R package scMetabolism with the ‘AUCell’ method.

Upon pathogen infection, monocytes undergo immune and metabolic reprogramming to fulfill their roles in combating infections. To further explore why pro-inflammatory monocytes are deeply involved in the PANoptosis process in COVID-19, we analyzed their immune and metabolic characteristics. In COVID-19, compared to other monocyte subpopulations, pro-inflammatory monocytes exhibited significant upregulation of pathways related to cell chemotaxis, cell migration, signal transduction and viral infection (Figure 5A and B). In the relatively early stage of post-ICU admission (T1), pro-inflammatory monocytes displayed heightened metabolic processes (Figure 5C). In the late stage of post-ICU admission (T2), pro-inflammatory monocytes displayed significant upregulation of pathways associated with cell differentiation and immune defense (Figure 5D). The different predominantly activated pathways of pro-inflammatory monocytes in the early and late stages of post-ICU admission indicated that the immune function of pro-inflammatory monocytes dynamically changed along with the infection course.

We further explored the immune characteristics of pro-inflammatory monocytes in COVID-19 in comparison to healthy controls. The pro-inflammatory monocytes in COVID-19 exhibited significant activation of pathways related to viral replication and antiviral immune responses, such as the response to type I interferon and NOD-like receptor signaling pathways (Figure 5E and F).

Concerning the metabolic characteristics of pro-inflammatory monocytes in COVID-19, alterations were observed notably in several metabolic pathway categories. These include changes in carbohydrate metabolism, lipid metabolism, amino acid metabolism, and metabolism of cofactors and vitamins (Figure 5G and H, Supplementary Figure 8A and B). These changes in metabolic landscape in pro-inflammatory monocytes in COVID-19 create suitable conditions for them to undergo PANoptosis.

In addition, dexamethasone treatment partially reversed the metabolic disturbances in COVID-19 (Figure 5G and H, Supplementary Figure 8A and B), indicating that dexamethasone could execute its therapeutic effects by modulating the disorderly metabolic patterns.

### PANoptosis predicted the ICU admission and progression in hospitalized COVID-19 patients

**Figure 6:**
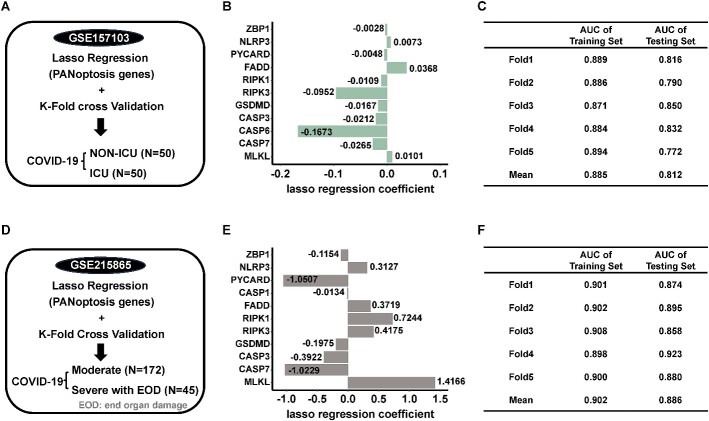
PANoptosis predicted the ICU admission and outcome of hospitalized COVID-19 patients. (**A**) Workflow depicting the analysis of the bulk RNA seq data from GSE157103. Thirteen PANoptosis genes (ZBP1, NLRP3, PYCARD, CASP1, CASP8, FADD, RIPK1, RIPK3, GSDMD, CASP3, CASP6, CASP7, MLKL) were used to construct a lasso regression model. *K*-fold cross-validation (*k* = 5, times = 5) was performed to assess the stability and generalizability of the model. (**B**) The mean lasso regression coefficient of PANoptosis genes included in the lasso regression model during a 5-fold cross-validation of bulk RNA-seq data from GSE157103. (**C**) Cross-validation results for lasso regression models with the 13 PANoptosis genes. The table presents cross-validation results for lasso regression models constructed using the 13 PANoptosis genes from the bulk RNA-seq dataset GSE157103. It contains three columns: Fold, AUC (Area Under the Curve) of Training Set and AUC of Testing Set. The ‘Fold’ column lists the names of the five individual folds (Fold 1 to Fold 5), and the ‘Mean’ row represents the average across these folds. The ‘AUC of Training Set’ column contains the mean AUC values calculated for the training sets of each fold, while the ‘Mean’ row displays the overall mean training AUC value. Similarly, the ‘AUC of Testing Set’ column contains the mean AUC values computed for the testing sets of each fold, and the ‘Mean’ row provides the overall mean testing AUC value. (**D**) Workflow depicting the analysis of the bulk RNA seq data from GSE215865. Thirteen PANoptosis genes (ZBP1, NLRP3, PYCARD, CASP1, CASP8, FADD, RIPK1, RIPK3, GSDMD, CASP3, CASP6, CASP7, MLKL) were used to construct a lasso regression model. *K*-fold cross-validation (*k* = 5, times = 5) was performed to assess the stability and generalizability of the model. (**E**) The mean lasso regression coefficient of PANoptosis genes included in the lasso regression model during a 5-fold cross-validation of bulk RNA-seq data from GSE215865. (**F**) Cross-validation results for lasso regression models with the 13 PANoptosis genes. The table presents cross-validation results for lasso regression models constructed using the 13 PANoptosis genes from the bulk RNA-seq dataset GSE215865. It contains three columns: Fold, AUC (Area Under the Curve) of Training Set and AUC of Testing Set. The ‘Fold’ column lists the names of the five individual folds (Fold 1 to Fold 5), and the ‘Mean’ row represents the average across these folds. The ‘AUC of Training Set’ column contains the mean AUC values calculated for the training sets of each fold, while the ‘Mean’ row displays the overall mean training AUC value. Similarly, the ‘AUC of Testing Set’ column contains the mean AUC values computed for the testing sets of each fold, and the ‘Mean’ row provides the overall mean testing AUC value.

Finally, to investigate the diagnostic potential of PANoptosis for predicting the disease outcome of COVID-19, we analyzed two bulk RNA-seq datasets compromising hospitalized COVID-19 patients (Figure 6A and D). We constructed lasso regression models using 13 PANoptosis genes and performed repeated *k*-fold cross-validation to evaluate their performance.

**Figure 7:**
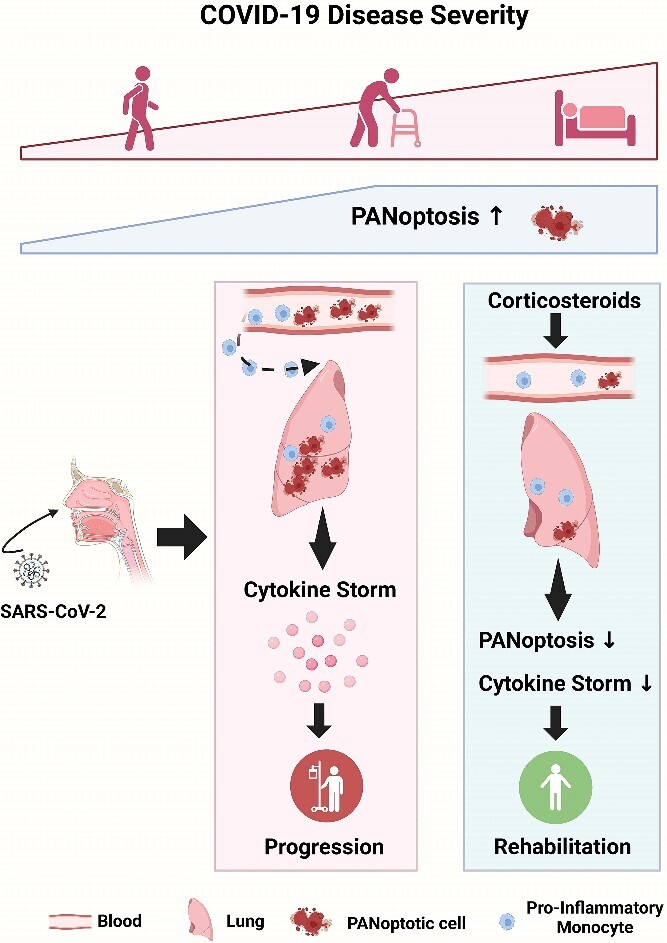
Schematic of the mechanisms by which PANoptosis mediates pathogenesis in COVID-19 (created in https://www.biorender.com/). PANoptosis mediates cytokine storms and multi-organ injuries in COVID-19. Elevated levels of PANoptosis were observed in COVID-19 compared to healthy controls, with the extent of PANoptosis positively correlated with disease severity. PANoptosis reached its peak in moderate COVID-19 cases, followed by a decrease in severe patients, possibly due to immune exhaustion in the advanced stages of the disease. Pro-inflammatory monocytes emerged as one of the primary sites of PANoptosis in COVID-19, migrating from the bloodstream to the lungs to execute immune defense functions during infection. In addition, corticosteroid treatments were found to potentially alleviate excessive inflammation in COVID-19 by mitigating PANoptosis.

The first model included 11 PANoptosis genes (ZBP1, NLRP3, PYCARD, FADD, RIPK1, RIPK3, GSDMD, CASP3, CASP6, CASP7, MLKL) (Figure 6B). This model effectively distinguished between patients admitted to the ICU and those who were not, as demonstrated by a receiver operating characteristic (ROC) analysis. The PANoptosis signature achieved a mean area under the curve (AUC) value of 0.885 in the training set and a mean AUC value of 0.812 in the testing set (Figure 6C).

Similarly, the second model consisted of 11 PANoptosis genes (ZBP1, NLRP3, PYCARD, CASP1, FADD, RIPK1, RIPK3, GSDMD, CASP3, CASP7, MLKL) (Figure 6E). This model effectively distinguished between moderate COVID-19 patients and severe patients with end-organ damage, with a mean AUC value of 0.902 in the training set and a mean AUC value of 0.886 in the testing set (Figure 6F).

To further demonstrate the classification ability of PANoptosis genes, we randomly selected an equal number of genes from the bulk RNA-seq datasets and constructed lasso regression models with them. We then performed *k*-fold cross-validation (Supplementary Figure 9A–C, E–G). Notably, PANoptosis genes exhibited superior classification performance compared to randomly selected genes (Supplementary Figure 9D and H).

These results highlighted the potential of PANoptosis as a valuable indicator for predicting the need for ICU admission and disease outcomes in hospitalized COVID-19 patients.

## DISCUSSION AND CONCLUSION

PANoptosis has been previously implicated in host defense against SARS-CoV-2 and viral pathogenesis in COVID-19 [4, 14]. In this study, we systematically analyzed the transcriptome and cellular landscape of PANoptosis in the context of COVID-19. Our finding revealed that the degree of PANoptosis in bronchoalveolar lavage fluid (BALF) and peripheral blood mononuclear cells (PBMC) served as an indicator of COVID-19 severity. Utilizing single-cell transcriptomics, we identified pro-inflammatory monocytes as a prominent site of PANoptosis in COVID-19. Subsequent investigations shed light on the immune and metabolic characteristics of these pro-inflammatory monocytes. Notably, pro-inflammatory monocytes exhibited heightened activation of pathways associated with cell chemotaxis, cell migration, signal transduction and immune defense. Regarding the metabolic properties of pro-inflammatory monocytes in COVID-19, alterations were observed in several metabolic pathway categories, including carbohydrate, lipid and amino acid metabolism. Furthermore, our analysis illustrated that dexamethasone may alleviate inflammation in COVID-19 by attenuating PANoptosis. Finally, our study demonstrated that the PANoptosis-related genes held potential in predicting ICU admission and outcomes among hospitalized COVID-19 patients, offering a new perspective on COVID-19 prognosis.

Programmed cell death mechanisms are of critical importance in shaping disease trajectories and outcomes. PANoptosis, a recently emerging concept encompassing the intricate interplay of multiple PCD pathways (pyroptosis, apoptosis and necroptosis), holds great potential for advancing our understanding of disease mechanisms. PANoptosis has been implicated in the pathogenesis of multiple systemic diseases, including infectious diseases [14, 29–32], cancer [33–35], neurodegenerative diseases [36, 37] and inflammatory diseases [38–40]. PANoptosis mediates cytokine storm and multi-organ injuries in COVID-19 [4, 14]. Our study found that PANoptosis was upregulated in COVID-19 compared to the healthy controls, and the level of the key genes of PANoptosis is linearly correlated with disease severity. Interestingly, PANoptosis peaked in moderate COVID-19, and there was a decrease in severe patients. The decrease of PANoptosis in disease deterioration may be due to immune exhaustion in severe cases, resulting in a diminished capacity for PANoptosis activation. Moreover, PANoptosis dramatically diminished in both the BALF and PBMC of severe patients who unfortunately did not survive during hospitalization, suggesting a link between a worse clinical outcome and decreased PANoptosis in severe COVID-19 patients.

Numerous studies have underscored the prognostic potential of PANoptosis-related genes in various diseases, encompassing cancer [41–45], cardiovascular diseases [46] and autoimmune disease [47, 48]. However, the prognostic relevance of PANoptosis in infectious conditions has remained largely unexplored. In our investigation, we delved into the potential of the PANoptosis feature as a tool for identifying severe patients. Our findings illustrated that the lasso regression model, including PANoptosis genes, was capable of distinguishing severe COVID-19 patients from those non-severe hospitalized cases, achieving impressive AUC values of ~0.9. This suggested that PANoptosis holds promise as an indicator of disease progression and outcomes. A deeper understanding of PANoptosis could pave the way for more tailored and effective treatments, ultimately improving patient prognosis.

Since the outbreak of the COVID-19 pandemic, it has become increasingly evident that aberrant immune responses against SARS-CoV-2 constitute a central feature of disease pathogenesis, especially in severe cases [49]. Corticosteroids can modulate the immune response in a non-specific and general manner. Several randomized control trials have revealed that corticosteroid therapy is effective in reducing the severity and mortality of COVID-19 [50–52]. Corticosteroids have shown particular benefits in patients requiring either invasive mechanical ventilation or oxygen alone, as opposed to individuals not needing respiratory support [53]. These findings have prompted WHO to strongly recommend corticosteroids for hospitalized COVID-19 patients who need respiratory support [28]. Our analysis indicated that the administration of dexamethasone in severe COVID-19 led to a decrease in PANoptosis. Notably, the effectiveness of dexamethasone became more evident over a comparatively extended treatment period. Previous studies suggested that glucocorticoids protect monocytes from PCD by activating adenosine receptor A3 [54]. Moreover, glucocorticoids could suppress the synthesis of TNF-α and IFN-γ [55], which might be another mechanism to explain the alleviating PANoptosis in dexamethasone-treated patients as TNF-α and IFN-γ initiate PANoptosis in COVID-19 [4]. In addition, we found that dexamethasone partially reversed the metabolic disorders closely associated with the onset of PANoptosis in COVID-19. Dexamethasone exhibited a partial restorative effect on the disruptions observed in carbohydrate, lipid, amino acid, cofactor and vitamin metabolism. These findings suggested that dexamethasone inhibits PANoptosis through multiple mechanisms.

Monocytes mediate the host antimicrobial defense and pathogenesis of inflammatory diseases [27]. Inflammation increases the egress of monocytes from their generative bone marrow niche [27]. Pro-inflammatory monocytes in COVID-19 demonstrate a considerable activation of pathways related to cell chemotaxis and migration when contrasted to other monocyte subpopulations, providing favorable conditions for the recruitment of pro-inflammatory monocytes to the inflamed sites. Moreover, the significant augmentation of cytokine responses, signal transduction and viral infection pathways in pro-inflammatory monocytes support their crucial role in antiviral immunity. Interestingly, our analysis revealed dynamic changes in the functional pathways of pro-inflammatory monocytes during the disease course. In the early stage of post-ICU admission (T1), pro-inflammatory monocytes displayed heightened activity in metabolic processes, whereas in the late stage of the post-ICU admission (T2), pro-inflammatory monocytes exhibited notable activation of pathways involved in cell differentiation and immune defense. The distinct patterns of predominantly activated pathways in different stages of the disease course underscore the dynamic changes in pro-inflammatory monocyte functions along with the course of the disease. The pro-inflammatory monocytes in COVID-19 exhibit significant activation of pathways related to viral replication and antiviral immune responses, such as response to type I interferon and NOD-like receptor signaling pathways.

Regarding the metabolic landscape of pro-inflammatory monocytes in COVID-19, changes in carbohydrate, lipid, amino acid, cofactor and vitamin metabolism were observed. These metabolic reprogramming events play a crucial role in shaping immune responses and PCD scenarios throughout the disease course. The alterations in metabolic pathways of pro-inflammatory monocytes in COVID-19 shed light on the intricate interplay between metabolism and the immune response. The unique immune and metabolic state exhibited by pro-inflammatory monocytes forms the foundation for the argument that they serve as one of the primary sites of PANoptosis.

This study still has several limitations that need to be considered in our future work. Firstly, the relatively small sample size and insufficiently diversified sample types limit the generalizability of our findings. In this study, only BALF and PBMC samples were included in this study. Another limitation of our study concerns the heterogeneity among patients and the timing of sample collection. The patients exhibited variations in the timing of their clinical presentation, genetic background, underlying diseases, age and gender, which could potentially impact their transcriptional landscapes. In addition, the findings in this study cannot be utilized as guidance for clinical practice at this point. Further experiments are required to confirm the impact of PANoptosis on the pathogenesis of COVID-19.

**Key Points**

- PANoptosis is reported to engage in host defense against SARS-CoV-2 and viral pathogenesis in COVID-19. We systematically analyzed the transcriptome and cellular landscape of PANoptosis concerning COVID-19. The degree of PANoptosis in bronchoalveolar lavage fluid and peripheral blood mononuclear cells reflected the severity of COVID-19.
- Single-cell transcriptomics identified pro-inflammatory monocytes as one of the primary sites of PANoptosis in COVID-19. The study demonstrated the immune and metabolic characteristics of this group of pro-inflammatory monocytes. Pro-inflammatory monocytes exhibited heightened activation of pathways associated with cell chemotaxis, cell migration, signal transduction and immune defense. In the context of COVID-19, significant changes were noted in the metabolic characteristics of pro-inflammatory monocytes, affecting various metabolic pathways such as carbohydrate, lipid and amino acid metabolism.
- The analysis illustrated that dexamethasone was likely to alleviate inflammation in COVID-19 by mitigating PANoptosis.
- The study showed that the PANoptosis-related genes could predict the ICU admission and outcomes of hospitalized COVID-19 patients, which may provide a new perspective on COVID-19 prognosis.

## Supplementary Material

sFig1_bbae124

sFig2_bbae124

sFig3_bbae124

sFIG4_bbae124

sFig5_bbae124

sFig6_bbae124

sFIG7_bbae124

sFig8_bbae124

sFig9_bbae124

Confirmation_of_Publication_and_Licensing_Rights_of_Figure7_bbae124

## Data Availability

The scRNA-seq data for COVID-19 analysis in this paper were sourced from the Gene Expression Omnibus (GEO) database (https://www.ncbi.nlm.nih.gov/geo/) with accession number GSE145926 (BALF) and GSE157789 (PBMC-Dex). The dataset of PBMC was obtained from https://covid19cellatlas.org/. Bulk RNA-seq data utilized in this study were retrieved from the GEO database with accession numbers GSE157103 and GSE215865.

## Author Biographies

**Qingyuan Yang** Obtained a Bachelor's degree in Clinical Medicine from Shanghai Jiao Tong University in 2020. Currently pursuing a PhD in Internal Medicine at Shanghai Jiao Tong University. The research focus is on the pathogenic mechanisms and treatment of challenging pulmonary infections.

**Wanmei Song** Earned a Master's degree in Internal Medicine from Shandong University in 2022. Currently pursuing a PhD in Internal Medicine at Shanghai Jiao Tong University. The research focus is on the pathogenesis and intervention of pneumonia.

**Hanizaier Reheman** Currently pursuing a Master's degree in Internal Medicine at Shanghai Jiao Tong University. The primary research focus is on the resistance mechanisms of pneumonia-causing pathogens.

**Dan Wang** Currently pursuing a PhD in Internal Medicine at Shanghai Jiao Tong University. The main research focus is on the pathogenesis of leukemia.

**Jieming Qu** A doctoral supervisor at Shanghai Jiao Tong University. Long committed to the diagnosis, treatment, and mechanism research of challenging and critical pulmonary infections.

**Yanan Li** Assistant researcher at the Department of Respiratory and Critical Care Medicine, Ruijin Hospital affiliated with Shanghai Jiao Tong University School of Medicine. The research focus is on pulmonary infections and immunity.
