# Supplementary figures and images for: PANoptosis, an indicator of COVID-19 severity and outcomes
Source: Brief Bioinform. 2024 Mar 28;25(3):bbae124. doi: 10.1093/bib/bbae124 (PMC10981763; doi:10.1093/bib/bbae124)

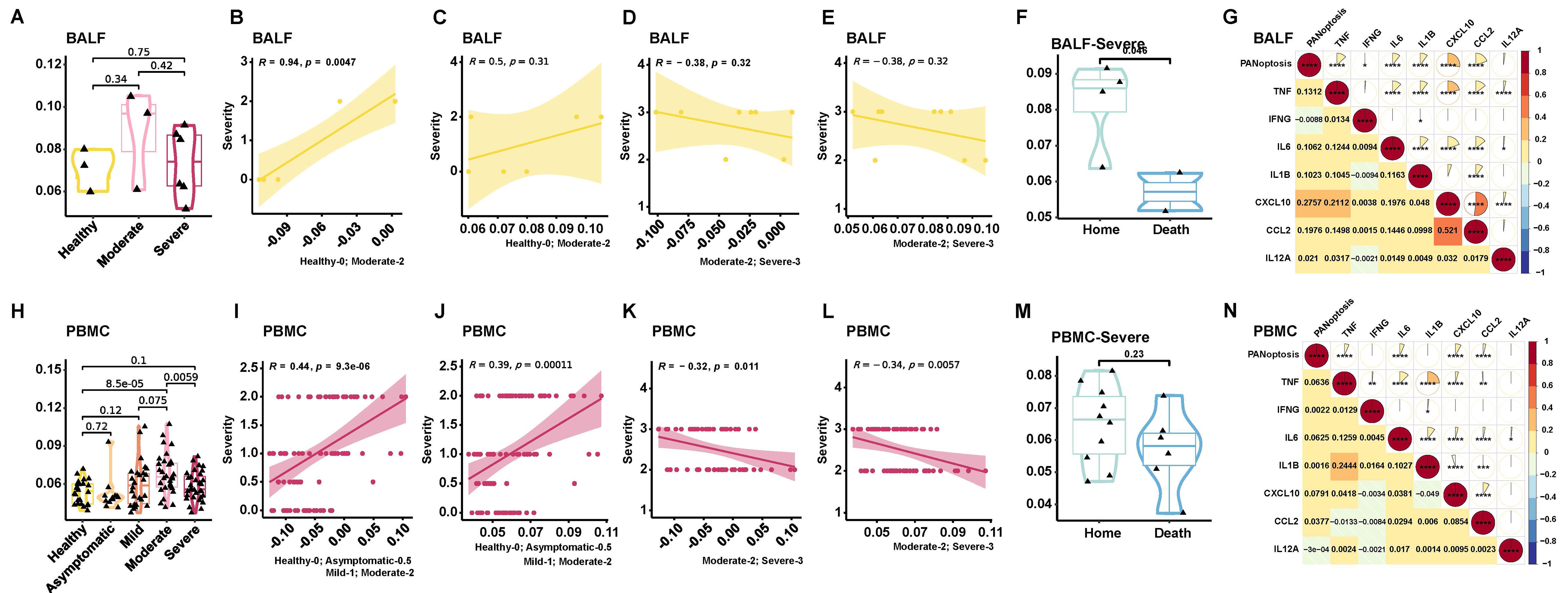

Supplement: sFig1_bbae124 [file sfig1_bbae124.jpeg]

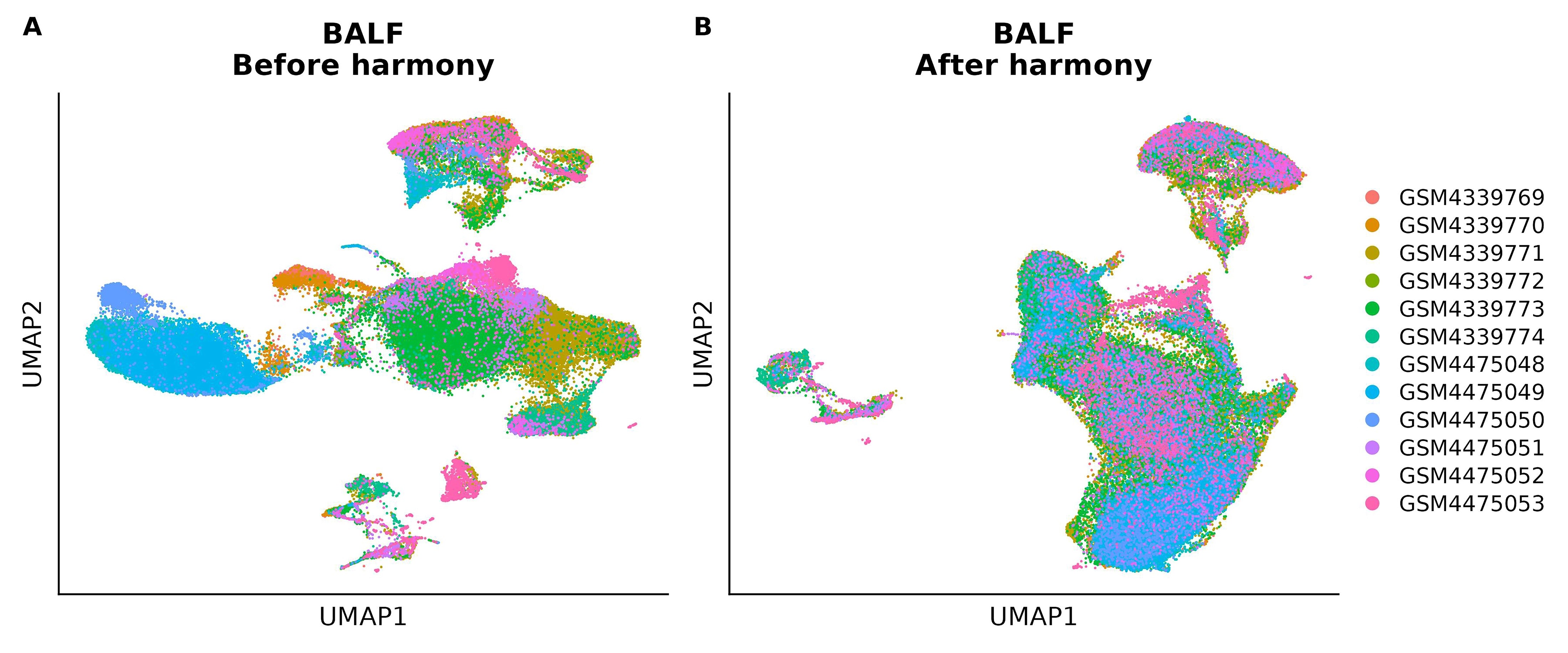

Supplement: sFig2_bbae124 [file sfig2_bbae124.jpeg]

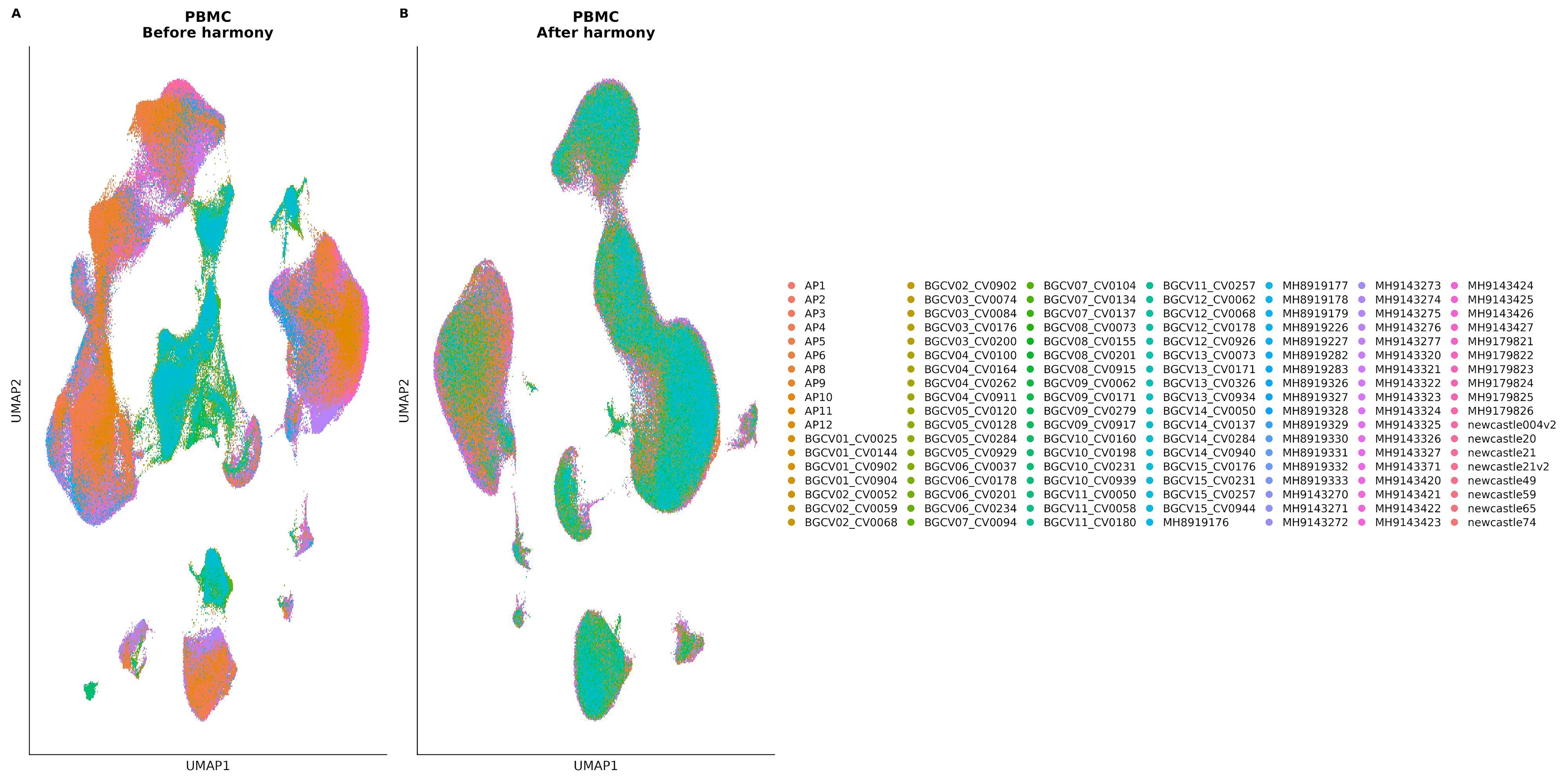

Supplement: sFig3_bbae124 [file sfig3_bbae124.jpeg]

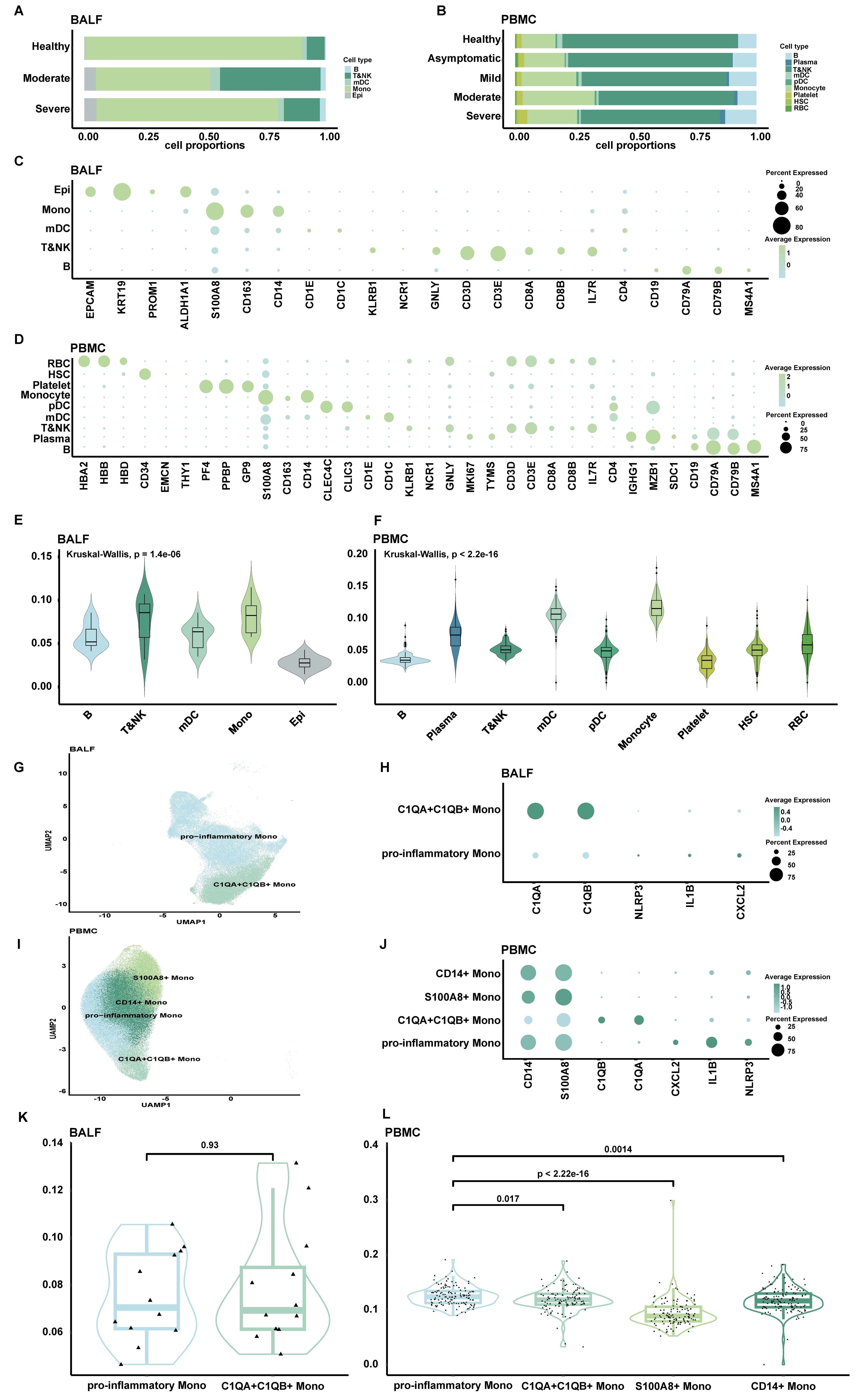

Supplement: sFIG4_bbae124 [file sfig4_bbae124.jpeg]

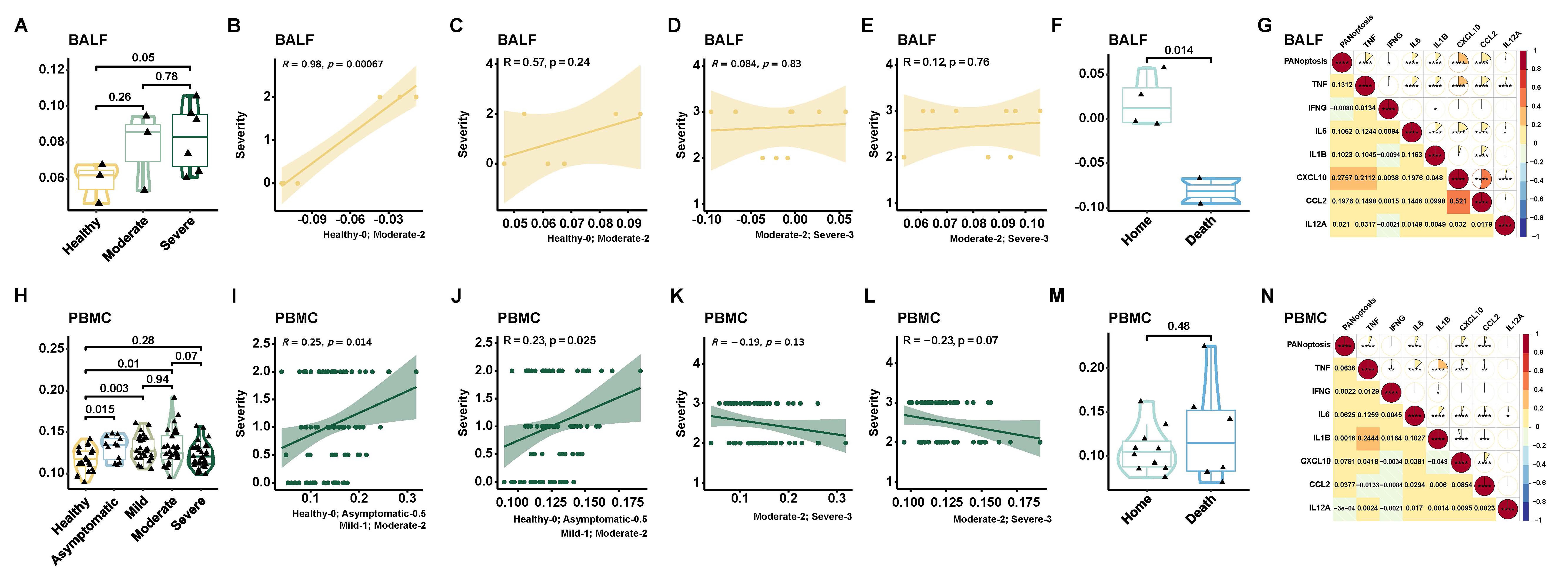

Supplement: sFig5_bbae124 [file sfig5_bbae124.jpeg]

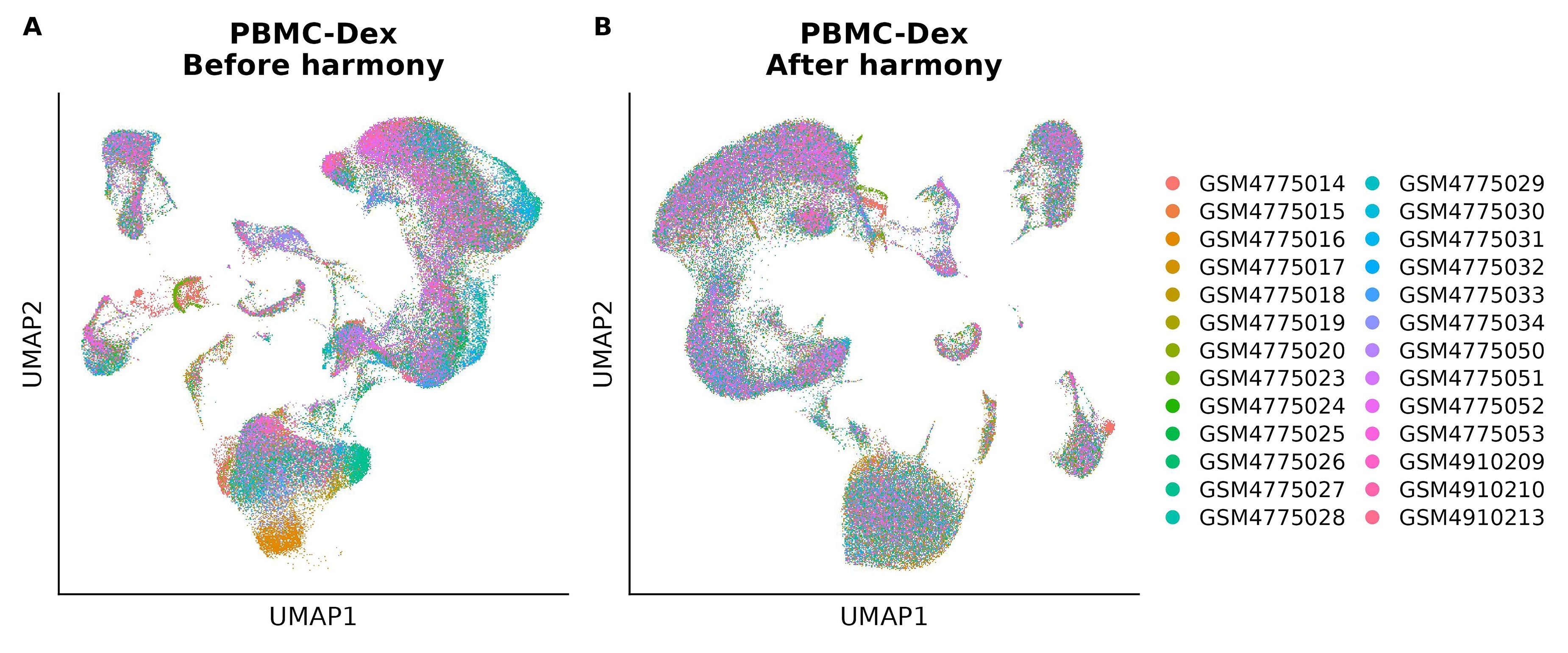

Supplement: sFig6_bbae124 [file sfig6_bbae124.jpeg]

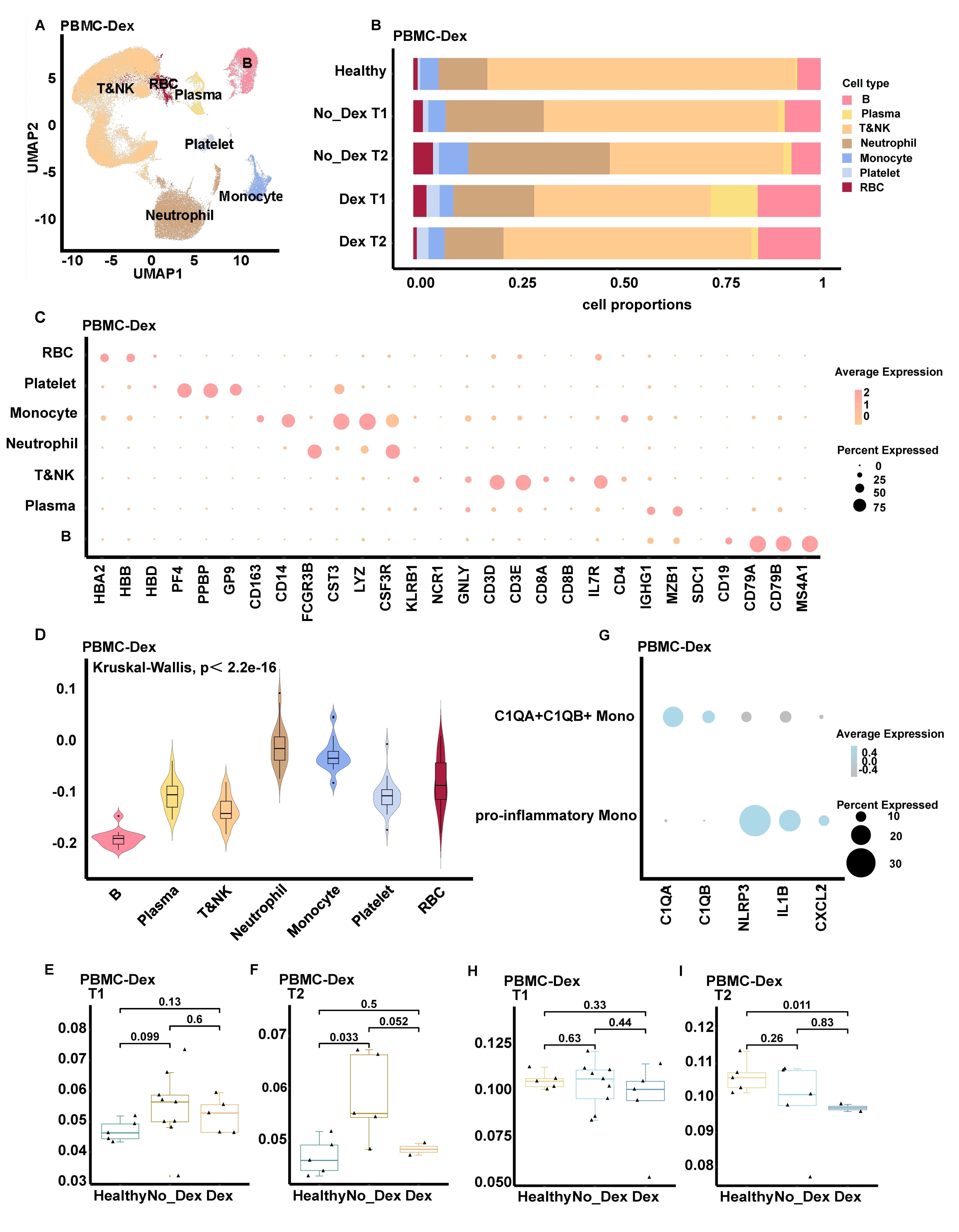

Supplement: sFIG7_bbae124 [file sfig7_bbae124.jpeg]

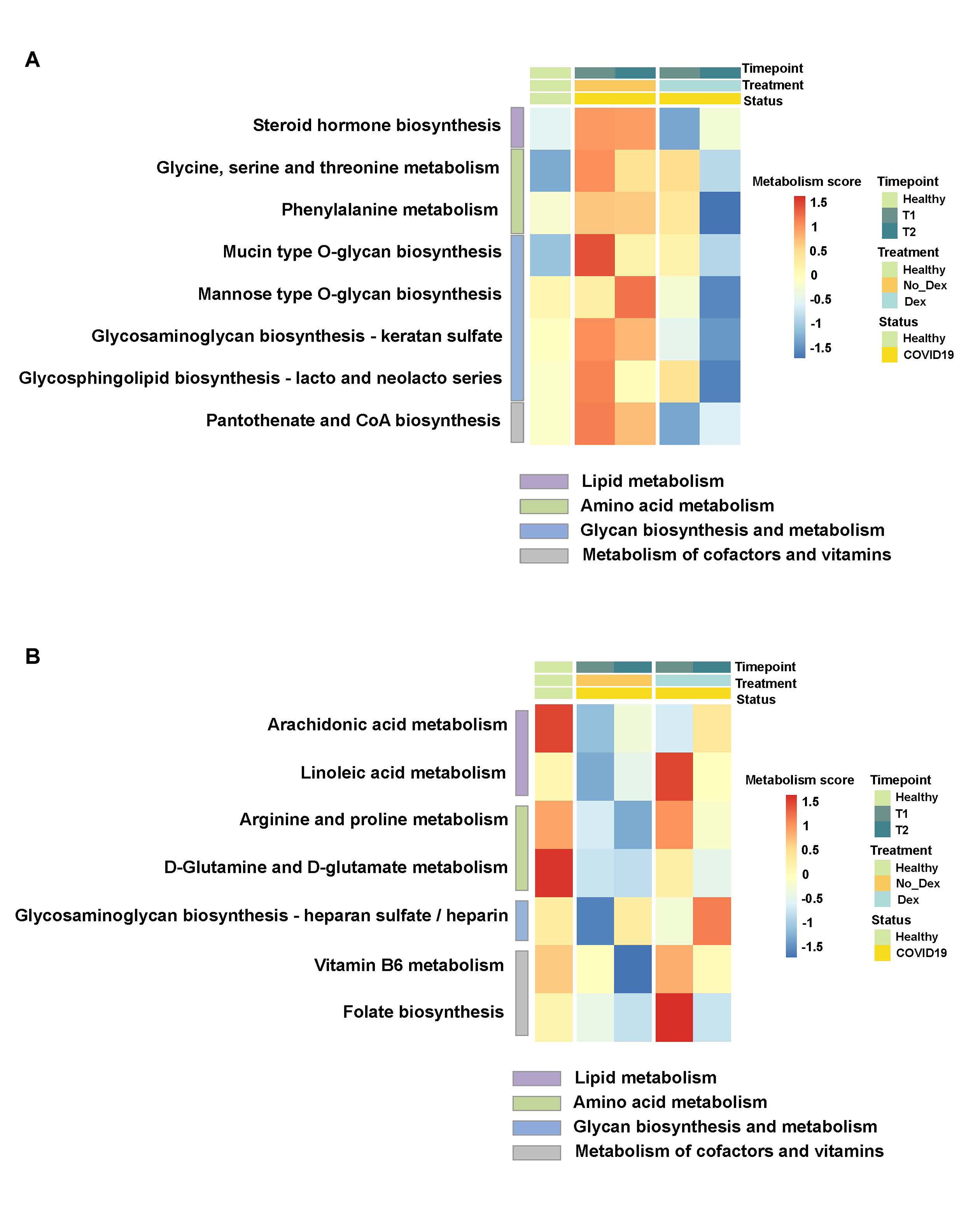

Supplement: sFig8_bbae124 [file sfig8_bbae124.jpeg]

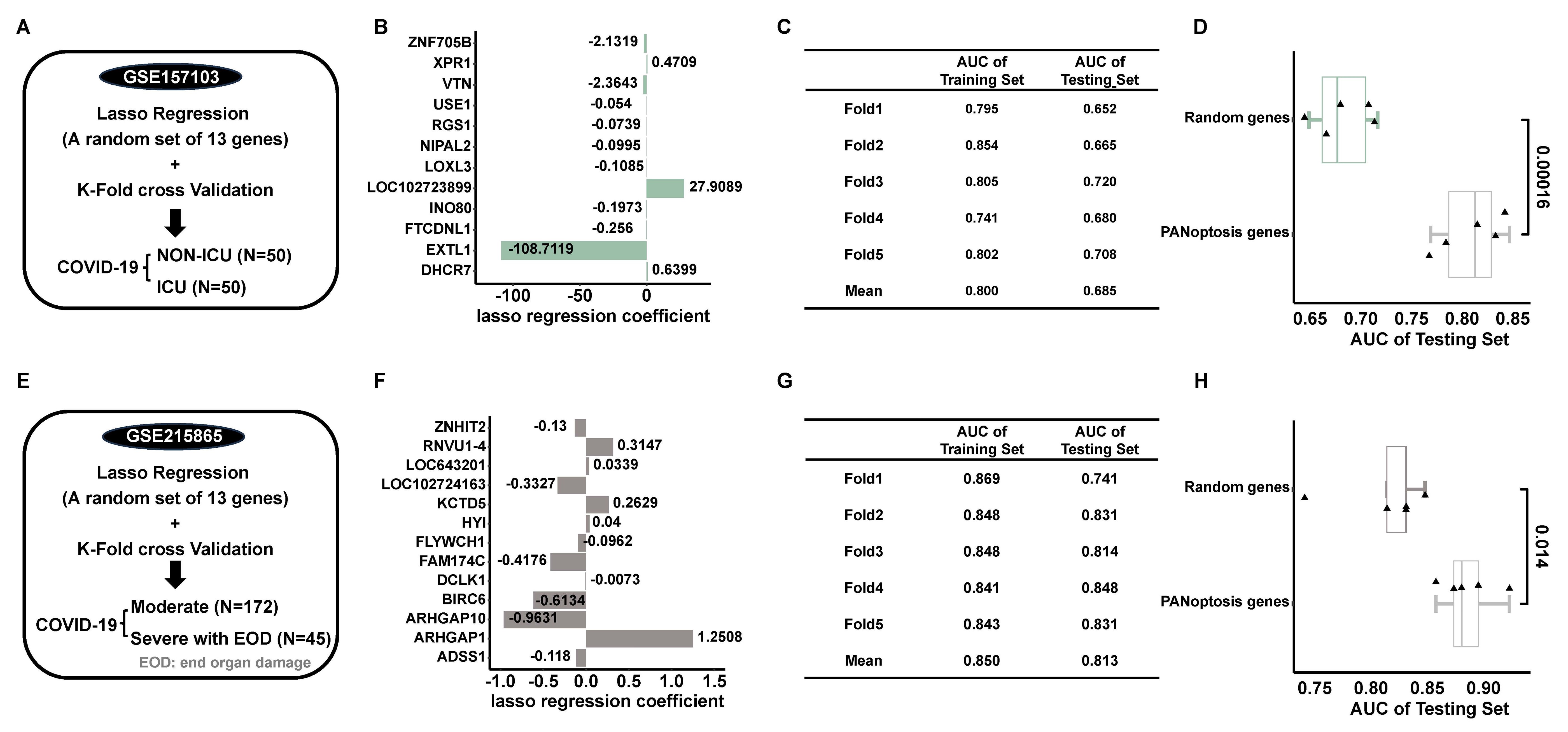

Supplement: sFig9_bbae124 [file sfig9_bbae124.jpeg]

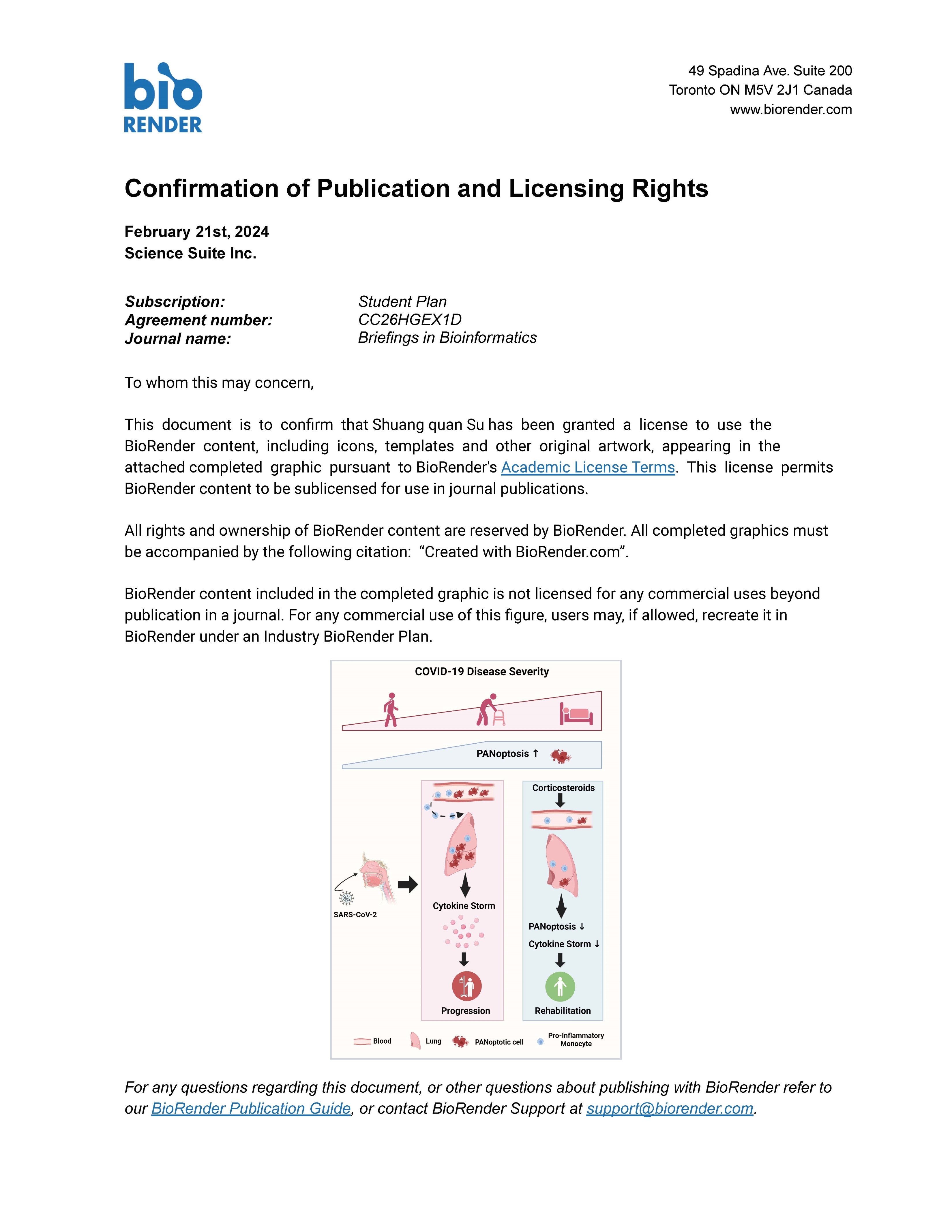

Supplement: Confirmation_of_Publication_and_Licensing_Rights_of_Figure7_bbae124 [file confirmation_of_publication_and_licensing_rights_of_figure7_bbae124.jpeg]
